# Author Correction: Nanofocusing of hard X-ray free electron laser pulses using diamond based Fresnel zone plates

**DOI:** 10.1038/s41598-020-62784-4

**Published:** 2020-04-08

**Authors:** C. David, S. Gorelick, S. Rutishauser, J. Krzywinski, J. Vila-Comamala, V. A. Guzenko, O. Bunk, E. Färm, M. Ritala, M. Cammarata, D. M. Fritz, R. Barrett, L. Samoylova, J. Grünert, H. Sinn

**Affiliations:** 1 Paul Scherrer Institut, CH-5232 Villigen, Switzerland; 2 SLAC National Accelerator Laboratory, Menlo Park, CA 94025 USA; 3 Department of Chemistry, University of Helsinki, Helsinki, FI-00014 Finland; 4 European Synchrotron Radiation Facility, F-38000 Grenoble, France; 5 European XFEL, D-22607 Hamburg, Germany

Correction to: *Scientific Reports* 10.1038/srep00057, published online 08 August 2011

A supplementary file containing supplementary material A and B is missing from this Article and is linked to this Author Correction.

## Supplementary information

Supplementary Figures.

