# Supplementary material for: Author Correction: Nanofocusing of hard X-ray free electron laser pulses using diamond based Fresnel zone plates
Source: Sci Rep. 2020 Apr 8;10:6282. doi: 10.1038/s41598-020-62784-4 (PMC7142139; doi:10.1038/s41598-020-62784-4)
Supplement: Supplementary file 1 — Supplementary Figures. [file 41598_2020_62784_MOESM1_ESM.doc]

**Nanofocusing of hard X-ray free electron laser pulses
using diamond based Fresnel zone plates**

**C. David, S. Gorelick, S. Rutishauser, J. Krzywinski, J. Vila-Comamala,
V. A. Guzenko, O. Bunk, E. Färm, M. Ritala, M. Cammarata, D. M. Fritz,
R. Barrett, L. Samoylova, J. Grünert, and H. Sinn**

**Supplementary Material**

**A: Characterization of a diamond FZP by evaluation of imprints**

In addition to the resolution tests on the iridium-filled diamond FZP described in the manuscript, we also carried out the corresponding investigations with a diamond FZP without Ir filling. The Supplementary Fig 1 shows the analysis of the imprint diameters. The results are very similar to those presented in Fig 5 of the manuscript. The main difference between the two types of FZPs lies in the diffraction efficiency, which was almost 4x lower during the measurements shown here. This difference was taken into account when normalizing the x-axis of Supplementary Fig 1a, and the y-axes of Supplementary Fig 1b. The choice of the attenuator settings was not well matched to the lower diffraction efficiency, resulting in fewer data points at high pulse energies and less accurate analysis of the focal spot. Nevertheless, the data shown in the Liu plot again seem to follow two different slopes, corresponding to a double-Gaussian spot profile as for the Ir-filled FZP. We find values for σ_1_, σ_2_, E_0,1_, and E_0,2_ that are very similar, leading to essentially the same FWHM value of 320 nm for the focal spot. Consistent with the lower diffraction efficiency, the peak fluence derived in Supplementary Fig 1b is approximately 7×10^3^ J/cm^2^, leading to a peak power of 1×10^17^ W/cm^2^.

**Supplementary Fig 1  Analysis of the nanofocus spot size of a diamond FZP**. (a) The squared imprint diameters as a function the logarithm of the pulse energy reveals two regions that can be approximated by straight lines corresponding to a double-Gaussian spot with sigma-values of 120 nm and 300 nm, respectively. (b) Beam profile derived from the same data set. The left y-axis shows the inverse pulse energies of the individual shots. The right y-axis is normalised such that the integral energy over the analytical double-Gaussian function is equal to the focused pulse energy *E_max_* = 1.3x10^-5^ J, when no attenuators are in the beam.

**B: Comparison of damage on Au FZPs with 10 Hz and 60 Hz repetition rate**

We carried out radiation damage tests on Fresnel zone plates (FZPs) made of gold at 8 keV photon energy and 1.2 mJ pulse energy of the LCLS source by comparing the scanning electron micrographs of FZPs that had received different numbers of pulses. In order to investigate a possible dose-rate effect, this experiment was performed at 10 Hz and 60 Hz repetition rate. The supplementary Fig 2 shows that the damage on the Au FZP structures is indeed stronger for the higher repetition rate. This supports the interpretation that the temperature rise caused by accumulation of heat over a certain number of shots depends on the time and thus the possibility of heat dissipation through radiation or conduction through the support membrane.

The apparent vertical stripes in the left images (1,000 pulses) are due to slight intensity variations of the beam profile. These were caused by diffraction on the aperture of slits situated 1.5 m upstream of the FZPs during the irradiation.


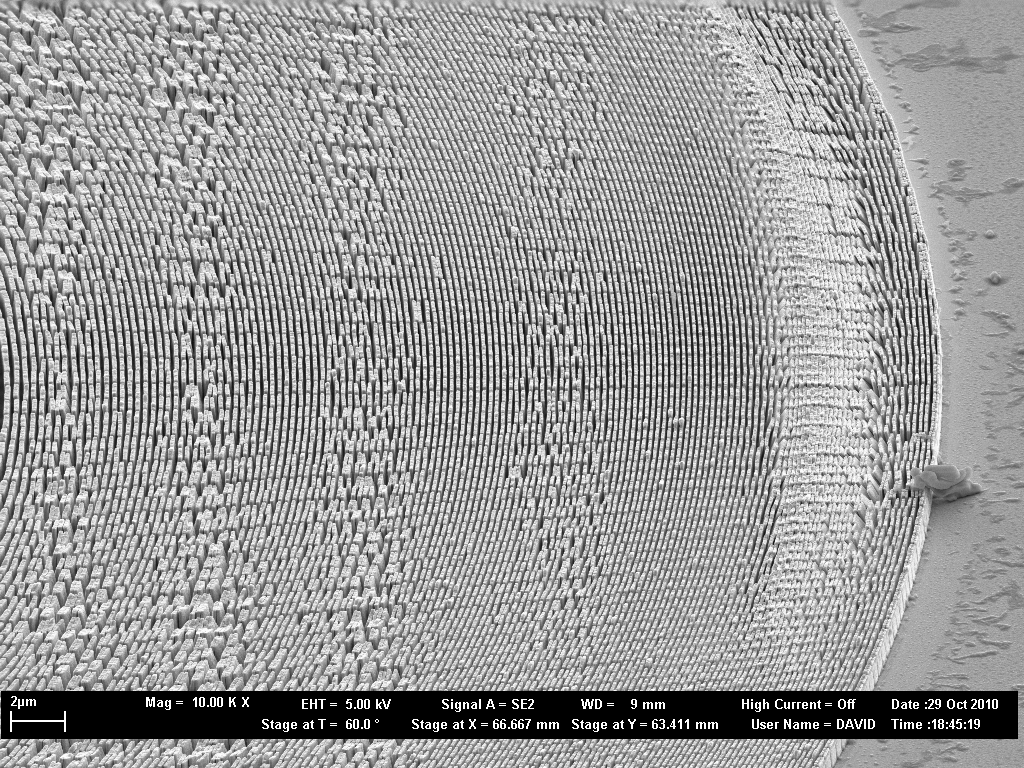

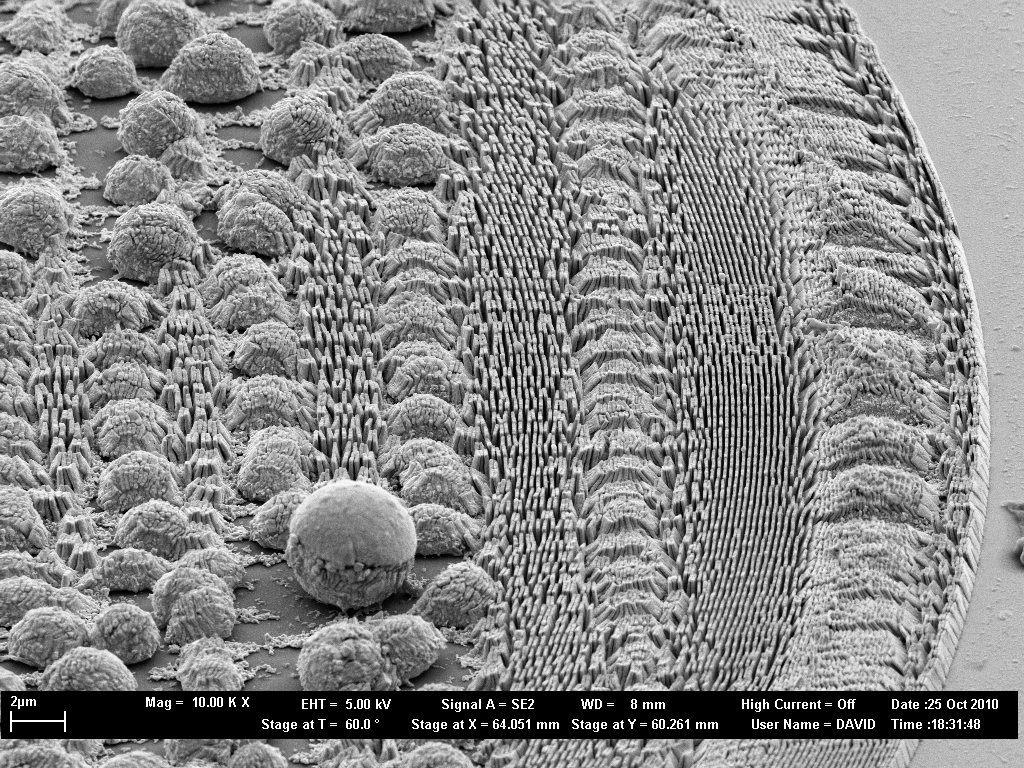


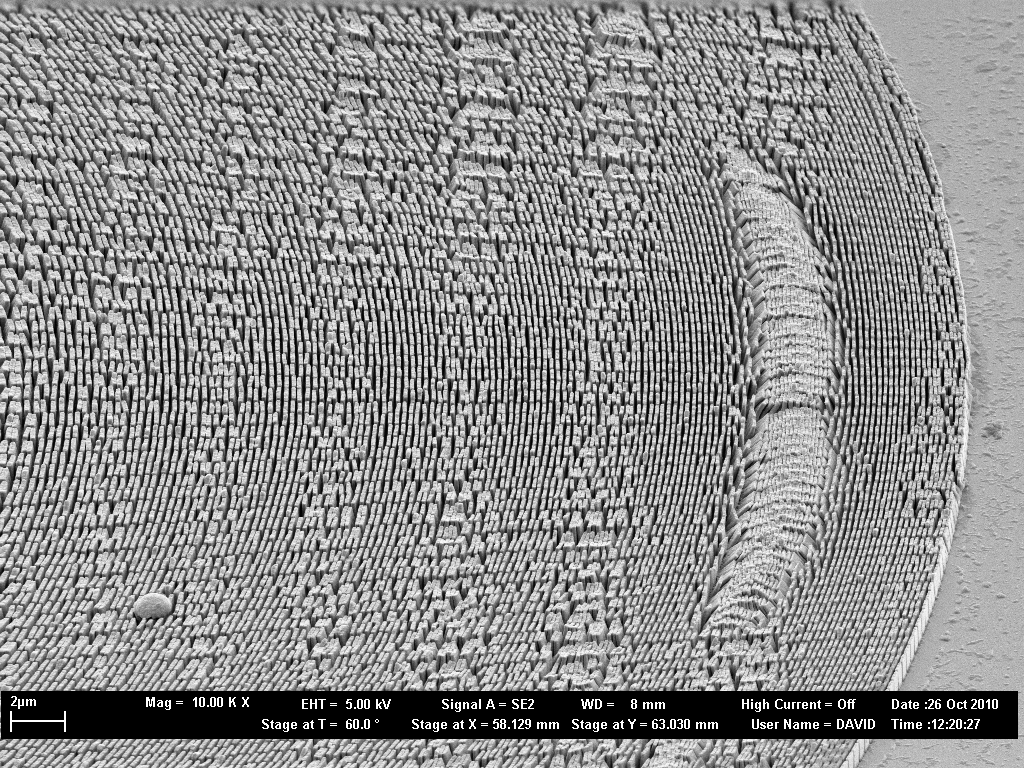

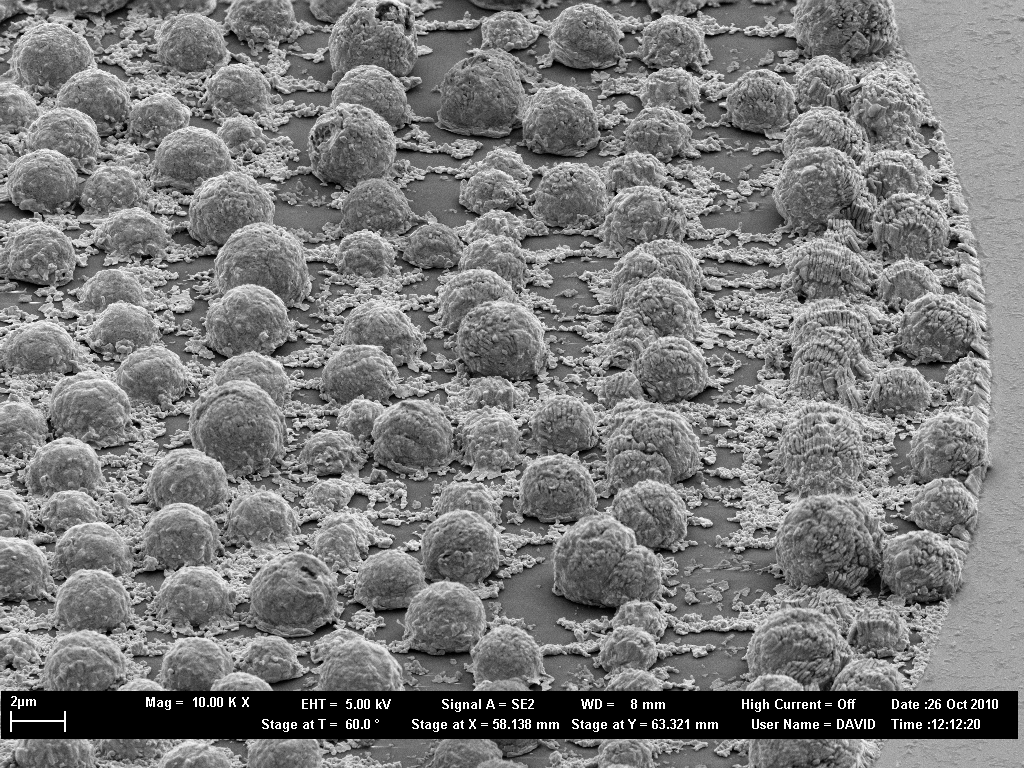


**Supplementary Fig 2  .** SEM images of identical FZPs with 1 µm high structures and an outermost zone width of 100 nm after irradiation. The top images show the FZPs that were irradiated at 10 Hz repetition rate (upper left: 1,000 pulses; upper right: 10,000 pulses). The lower images are identical to the ones shown in Fig. 1 of the manuscript and show the FZPs that were irradiated at 60 Hz repetition rate (lower left: 1,000 pulses; lower right: 10,000 pulses). The LCLS pulse power was 1.2 mJ. The scale bar is 2 µm, the viewing angle is 45°.
